# Effects of Fibrolytic Enzymes Alone or with Live Yeast on Rumen Microbiota and Fermentation During Grazing-to-Indoor Transition in Dairy Cows

**DOI:** 10.3390/life16040685

**Published:** 2026-04-18

**Authors:** Ignas Šilinskas, Ilma Tapio, Ingrida Monkevičienė, Kristina Musayeva, Hanna Huuki, Rūta Šilinskienė, Dovile Klupsaite, Elena Bartkiene, Aldona Baltušnikienė, Renata Japertienė, Vaidas Oberauskas, Rasa Želvytė

**Affiliations:** 1 Research Center for Digestive Physiology and Pathology, Department of Anatomy and Physiology, Lithuanian University of Health Sciences, Tilžės 18, LT-47181 Kaunas, Lithuania; ingrida.monkeviciene@lsmu.lt (I.M.); vaidas.oberauskas@lsmu.lt (V.O.); 2 Production Systems, Natural Resources Institute Finland (Luke), Myllytie 1, FI-31600 Jokioinen, Finland; 3 Department of Anatomy and Physiology, Lithuanian University of Health Sciences, Tilžės 18, LT-47181 Kaunas, Lithuania; ruta.silinskiene@lsmu.lt; 4 Department of Agricultural Sciences, University of Helsinki, Koetilantie 5, FI-00790 Helsinki, Finland; 5 Welfare Sciences, Faculty of Social Sciences, Tampere University, Kalevantie 5, FI-33100 Tampere, Finland; 6 Institute of Animal Rearing Technologies, Lithuanian University of Health Sciences, Mickeviciaus Str. 9, LT-44307 Kaunas, Lithuania; dovile.klupsaite@lsmu.lt (D.K.); elena.bartkiene@lsmu.lt (E.B.); 7 Department of Biochemistry, Faculty of Medicine, Lithuanian University of Health Sciences, Mickeviciaus Str. 9, LT-44307 Kaunas, Lithuania; aldona.baltusnikiene@lsmu.lt; 8 Department of Animal Breeding, Faculty of Animal Sciences, Lithuanian University of Health Sciences, Tilžės 18, LT-47181 Kaunas, Lithuania; renata.japertiene@lsmu.lt

**Keywords:** rumen microbiota, fibrolytic enzymes, live yeast, rumen fermentation, nitrogen metabolism

## Abstract

Rumen microbial fermentation plays a central role in nutrient utilization and milk production in dairy cows. This study evaluated the effects of supplementation with exogenous fibrolytic enzymes, alone or in combination with live yeast on rumen microbiota, fermentation characteristics, nitrogen-related metabolites, and production performance during the transition from outdoor grazing to indoor housing. Thirty Lithuanian Red dairy cows were assigned to control (CTR), enzyme (E), or enzyme plus yeast (YE) treatments across outdoor (OD) and transit (T) periods, while nine cows (three per group) were selected for rumen and microbiota analysis. Rumen bacterial communities were characterized using 16S rRNA gene sequencing, and functional parameters were evaluated using linear mixed-effects models. Supplementation resulted in selective changes in several bacterial genera, including *Blautia* spp., *WPS-2*, *Ruminococcus* spp., *Erysipelotrichaceae* UCG-009, *Sharpea* spp., uncultured *Bacteroidales*, and *Prevotellaceae* UCG-003, and was associated with alterations in fermentation patterns, particularly propionate concentration, and in nitrogen metabolism, including putrescine dynamics. The transition period significantly influenced microbial diversity and total bacterial abundance across treatments. Cows in the YE group maintained higher milk yield during the transition period. Overall, dietary supplementation modulated specific rumen metabolic responses and contributed to production stability without causing large-scale changes in overall microbial structure.

## 1. Introduction

Rumen microbial fermentation is fundamental to nutrient conversion and productive performance in dairy cattle, as it determines the production of volatile fatty acids (VFAs) and other metabolites that support energy metabolism and milk synthesis in the host [1,2]. Rumen function depends on the activity of a complex microbial community. Therefore, modern nutritional approaches focus not only on animal production performance but also on indicators of rumen function, such as fermentation patterns and nitrogen-related metabolites [1,2]. Accordingly, evaluating dietary interventions using both functional parameters and microbial community data can strengthen biological interpretation and enhance the relevance of findings for applied dairy nutrition [1,3]. In practical dairy systems, one of the major challenges is the transition between feeding and management conditions, such as the shift from outdoor grazing to indoor housing periods with dietary changes. These transitions modify the type and amount of substrates entering the rumen, feeding patterns, and the rumen environment, leading to changes in fermentation and microbial community structure [4,5]. Previous research studies have demonstrated that changes in diet and its management strongly influence the rumen microbial community. Such shifts may reduce microbial stability and alter fermentation efficiency, particularly during transition periods. Therefore, nutritional interventions that support fiber degradation and stabilize rumen function may help maintain consistent fermentation patterns and animal performance [6,7].

Exogenous fibrolytic enzymes have been proposed as a targeted dietary supplements to improve the utilization of plant structural carbohydrates by enhancing fiber degradation and increasing the availability of fermentable substrates [8,9]. By enhancing fiber degradation, these additives may alter fermentation end-products and improve the efficiency of nutrient utilization [8]. However, responses to enzyme supplementation are often inconsistent across studies. The effects appear to depend on factors such as enzyme activity and dose, diet composition and forage quality, and the physiological state of the animal [9,10,11]. The variability in responses to enzyme supplementation warrants evaluation under controlled feeding conditions, and its effects should be interpreted using relevant functional parameters rather than being assessed solely on the basis of overall performance outcomes [9,10]. Live yeast and yeast-derived products are commonly applied in dairy nutrition because they are thought to help maintain rumen stability and support microbial activity, particularly during challenging feeding conditions [12,13]. However, responses to yeast supplementation are not consistent. Effects vary depending on the strain used, dosage, diet, and stage of production, and benefits are not consistently observed under all management conditions [12,14]. Consequently, the potential value of combining exogenous enzymes with live yeast remains an open applied question: the combination could yield additive or non-additive effects depending on context, and any advantages may be reflected more clearly in functional metabolic signals than in broad community-wide metrics alone [12,13]. Therefore, the practical value of combining exogenous enzymes with live yeast remains unclear. From a scientific perspective, it is also uncertain whether their combined use results in additive benefits, no interaction, or even antagonistic effects. Such effects may become most apparent during periods of major dietary transition, when shifts in substrate supply challenge rumen microbial stability, alter fermentation patterns, and potentially influence production performance.

Therefore, the aim of the present study was to evaluate dietary supplementation with exogenous enzymes, alone or in combination with live yeast, on rumen microbiota and rumen functional indicators during the outdoor grazing and the transition in to indoor housing. We hypothesized that supplementation with exogenous enzymes, alone or in combination with live yeast, would modulate rumen functional responses and microbial community composition during the transition from outdoor grazing to indoor feeding, thereby influencing fermentation patterns and nitrogen-related metabolism, with potential effects on production performance.

## 2. Materials and Methods

### 2.1. Experimental Design and Animals

The experiment was conducted at a commercial organic dairy farm in the Panevėžys region, Lithuania. The study lasted 82 days and consisted of a grazing (outdoor, OD) period followed by a transition to indoor housing (T). All experimental procedures involving animals were approved by the State Food and Veterinary Service of Lithuania (approval No. G2-60).

The trial was designed as a controlled feeding experiment with three dietary treatments and repeated measurements across experimental periods. Lithuanian Red dairy cows were allocated to treatment groups using the principle of analogues based on parity, body condition score (BCS), milk yield, and physiological status at the beginning of the trial. All cows were multiparous and in early lactation (60–90 days in milk), ranging from the 2nd to 4th lactation. Body condition score ranged from 2.75 to 3.00, and average milk yield ranged from 29 to 34 kg/day. Only clinically healthy cows were included, with somatic cell counts below 200 × 10^3^ cells/mL. No significant differences in these parameters were observed among the experimental groups at the start of the trial. During the grazing period, cows were loose-housed and grazed pasture for 6 h daily (11:00–17:00) in addition to receiving the total mixed ration (TMR). During the transition period, cows were kept loose-housed indoors and fed TMR exclusively. All cows were milked twice daily at 06:00 and 18:00.

Each treatment group consisted of 10 cows (total *n* = 30) receiving one of the following diets: a control diet without feed additives (CTR), a diet supplemented with exogenous enzymes (E), or a diet supplemented with exogenous enzymes combined with live yeast (YE). Milk yield and milk composition were recorded daily throughout both experimental periods. For rumen-related analyses, three cows per group were selected according to the analogue principle from the larger cohort. All selected animals were multiparous Lithuanian Red dairy cows with available production records from previous lactations. These animals were representative of their respective groups and did not differ significantly in baseline characteristics relevant to the experimental design.

### 2.2. Animal Feeding and Dietary Treatments

Diets were formulated to meet the nutritional and energy requirements of lactating dairy cows according to their physiological status and milk production level of approximately 33 kg per day. Throughout the experiment, all cows received a total mixed ration (TMR) providing approximately 20 kg of dry matter per cow per day. During the grazing period, cows received pasture herbage during daily grazing (11:00–17:00), composed mainly of timothy (*Phleum pratense*), red clover (*Trifolium pratense*), white clover (*Trifolium repens*), and lucerne (*Medicago sativa*), in addition to the total mixed ration (TMR). During the indoor period, cows were fed TMR exclusively. The TMR was offered twice daily at 07:00 and 17:00 and prepared by mixing all feed components for 10 min in a feed mixer to ensure homogeneity. Fresh drinking water was provided ad libitum throughout both experimental periods. The ingredient and chemical composition of the experimental diets are presented in Table 1.

The CTR group received the basal TMR. The enzyme-supplemented diet (group E) contained a fungal enzyme complex derived from *Trichoderma reesei*, providing endo-β-xylanase (3.7 × 10^5^ U/cow per day), endo-cellulase (4.5 × 10^5^ U/cow per day), and endo-β-glucanase (1.2 × 10^5^ U/cow per day) (Vilzim^®^NSP, “Biorro”, Vilnius, Lithuania). In the yeast + enzyme treatment (group YE), the same enzyme doses were applied in combination with live yeast (Saccharomyces cerevisiae CNCM I-1077) (Levucell SC, Lallemand Animal Nutrition, Blagnac, France) at a dose of 1.0 × 10^10^ CFU per cow per day. Total mixed ration (TMR) was prepared separately for each treatment group, with feed additives incorporated according to the number of cows to achieve the intended dose per cow. Additives were uniformly applied and mixed for 10 min prior to feeding to ensure homogeneous distribution. Cows were group-fed within their respective treatment groups; therefore, although individual intake was not measured, the average additive intake per cow corresponded to the intended dosage.

### 2.3. Sampling Strategy and Experimental Timeline

The experimental timeline was structured to capture both baseline conditions and temporal responses to dietary treatments during the grazing outdoor period (OD) and the subsequent transition period (T) to indoor housing.

Baseline samples were collected on day 0 (OD-0) prior to the initiation of dietary supplementation, then on day 31 (OD-31) and at the end of the grazing phase on day 62 (OD-62) to assess mid-term and cumulative treatment effects under outdoor conditions. Following the transition to indoor housing, sampling was performed on day 72 (T-72) and day 82 (T-82), corresponding to 10 and 20 days after housing transition, respectively, in order to evaluate short-term adaptation responses to indoor management.

Rumen fluid (approximately 1 L) was obtained using a rumen probe (Ruminator, Profs-Products, Wittibreut, Germany) from the caudoventral region of the rumen approximately 2 h before the morning feeding to minimize diurnal variation associated with feed intake. A subsample of 200 mL (in triplicate) for volatile fatty acid (VFA), biogenic amines and nitrogen analysis was stored at −20 °C until laboratory testing. For microbial analysis, 2 mL aliquots (in triplicate) were transferred into sterile cryogenic tubes, snap-frozen in liquid nitrogen, and stored at −80 °C until DNA extraction.

Fecal samples (2 L per cow) were collected immediately after rumen content sampling by obtaining them directly from the rectum of the same cows. After the samples had been collected, in vivo feed digestibility was analyzed.

Milk samples were taken during morning milking from all experimental cows (*n* = 30) on the planned sampling days. The milk sample was taken by following the control milk sampling rules [15].

### 2.4. Rumen Fermentation and Nitrogen Analyses

The pH of freshly collected rumen fluid was measured immediately after sampling using a portable pH meter (Twin pH meter, Horiba, Kyoto, Japan) following the manufacturer’s instructions. Immediate pH determination was performed to avoid post-sampling alterations associated with microbial activity and gas exchange. Volatile fatty acid (VFA) concentrations in rumen fluid were determined by gas chromatography according to the methodology described by Bureenok et al. [16]. Acetate, propionate, isobutyrate, butyrate, valerate, methyl-valerate, caproate, and enanthate concentrations were quantified (mmol/L) using a GC-2010 Plus gas chromatograph equipped with a mass spectrometry detector (GCMS-QP2010; Shimadzu Corp., Kyoto, Japan).

Biogenic amines, including tryptamine, phenylethylamine, putrescine, cadaverine, histamine, tyramine, spermidine, and spermine, were analyzed following the method procedures developed by Ben-Gigirey et al. [17] and are described by Bartkiene et al. [18]. Biogenic amines were analyzed as indicators of rumen nitrogen metabolism to provide additional information on protein degradation pathways and microbial metabolic responses under different dietary conditions, as well as in response to exogenous fibrolytic enzymes and live yeast.

Total nitrogen (TN) and ammonia nitrogen (NH_3_–N) concentrations in rumen fluid were determined according to the method described by Stern and Endres and expressed as mg/dL [19].

### 2.5. Rumen Contents DNA Analysis

DNA was isolated from 600 µm rumen contents following the protocol described by Ruis et al. [20]. The amount of rumen bacteria was estimated using quantitative real-time PCR (qPCR) by quantifying rRNA gene copy numbers in 1 ng of extracted DNA as described by Huuki et al. [21]. Briefly, total bacterial abundance was quantified using universal bacterial primers 520F (AGCAGCCGCGGTAAT) and 799R (CAGGGTATCTAATCCTGTT) as previously described by Edwards et al. [22].

qPCR reactions were performed using SYBR Green chemistry (PowerUp™ SYBR Green PCR Master Mix, Thermo Fisher Scientific, Waltham, MA, USA) on a ViiA™ 7 Real-Time PCR System (Applied Biosystems, Waltham, MA, USA). Each reaction was carried out in a final volume of 20 µL, containing 10 µL of SYBR Green master mix, 0.3 µL of each primer (10 µM), molecular biology grade water, and 2 µL of template DNA (20 ng per reaction). All samples were analyzed in triplicate, and non-template controls were included in each run.

Thermal cycling conditions consisted of an initial denaturation at 95 °C for 10 min, followed by 40 cycles of denaturation at 95 °C for 15 s and annealing/extension at 60 °C for 1 min. A melt curve analysis was performed at the end of each run to confirm amplification specificity.

Quantification was based on cycle threshold (Ct) values and calculated relative to calibration curves generated using quality control standards to run under identical PCR conditions. Results were expressed as 16S rRNA gene copy numbers per ng of genomic DNA.

### 2.6. 16S rRNA Gene Sequencing and Bioinformatic Analysis

Bacterial and archaeal community composition in rumen contents was assessed by sequencing the V4 region of the 16S rRNA gene using the universal primers 515F and 806R [23]. Amplicon libraries were prepared according to the Illumina 16S Metagenomic Sequencing Library Preparation protocol (Illumina, San Diego, CA, USA) and described by Huuki et al. [21].

Sequencing was performed on an Illumina MiSeq platform (Functional Genomics Centre, University of Turku, Turku, Finland) using paired-end chemistry (2 × 250 bp). Demultiplexing and adapter trimming were conducted by the sequencing service provider. Raw sequencing data were processed using QIIME 2 (version 2021.8.0) [24]. Paired-end reads were quality filtered, denoised, merged, and checked for chimeras using the DADA2 plugin, resulting in the generation of amplicon sequence variants (ASVs) [25]. Taxonomic assignment of bacterial and archaeal sequences was performed using a naïve Bayes classifier trained on the SILVA reference database (release 138, NR99), trimmed to the V4 [26]. Low-abundance features and singletons were removed prior to downstream analyses. Although both bacterial and archaeal communities were targeted, subsequent analyses focused primarily on bacterial community composition; archaeal taxa were retained and reported where relevant among the most abundant families.

### 2.7. Milk Yield and Milk Composition Analysis

Milk yield was recorded daily for all cows throughout the grazing and indoor periods using the milking system scales integrated into the DeLaval milking system (DeLaval, Tumba, Sweden), with data automatically recorded into the DelPro management software (version 5.0; DeLaval, Tumba, Sweden). Milk samples for compositional analysis were collected during the morning milking in accordance with official milk recording procedures [15]. Samples were submitted to an accredited laboratory (“Pieno tyrimai”, Kaunas, Lithuania) for analysis. Milk fat, protein, lactose, were determined by mid-infrared spectroscopy using a LactoScope FTIR analyzer (Delta Instruments, Drachten, The Netherlands). Energy-corrected milk (ECM) values were calculated and provided by the same laboratory (“Pieno tyrimai”, Kaunas, Lithuania) using a standardized equation:

ECM = milk yield (kg) × (0.122 × fat (%) + 0.077 × protein (%) + 0.253).

### 2.8. Physical Evaluation of Fecal Residues

The physical breakdown of feed was assessed using a fecal washing technique adapted from Hall [27]. A calibrated 2 L sample of fresh feces was collected directly from the rectum of each cow at predefined sampling points. Samples were obtained from the same cows used for rumen sampling (*n* = 9). The sample was processed using a Nasco Digestion Analyzer (Nasco, Fort Atkinson, WI, USA) according to the manufacturer’s instructions, by rinsing with tap water for 5–10 min until the effluent was clear. The remaining undigested macro-residues were returned to a same 2 L container to determine their volume (*V*_residue_).

The Washable Fecal Fraction (*WFF*) was calculated as follows:

$$WFF(\%)=\left(\frac{V_{initial}-V_{residue}\;}{V_{initial}}\right)\times 100$$

where is the initial fecal volume (2 L). This metric was used as an indicator of the physical effectiveness of the diet and particle breakdown in the rumen.

### 2.9. Statistical Analysis

Statistical analyses were performed using IBM SPSS Statistics (version 15; IBM Corp., Armonk, NY, USA), QIIME 2 (version 2021.8.0), and R (version 3.6.3; R Core Team, Vienna, Austria). Data were analyzed using linear mixed-effects models (LMM) to account for repeated measurements within individual cows across experimental periods. Data are presented as mean values with standard deviation (SD). When significant main effects or interactions were detected, additional period-specific comparisons were performed using one-way ANOVA within each experimental period. Statistical significance was declared at *p* < 0.05, and trends were discussed when 0.05 ≤ *p* < 0.10.

#### 2.9.1. Linear Mixed-Effects Modeling

For continuous response variables, including ruminal fermentation parameters, qPCR-derived bacterial abundance, milk yield, milk composition, and in vivo digestibility, linear mixed-effects models were applied. The general model included dietary treatment (Control, Enzyme, Yeast + Enzyme), experimental period (Outdoor, Transit), and their interaction (Group (G) × Period (P)) as fixed effects. Cow identity was included as a random effect to account for within-animal correlation due to repeated measurements.

The model structure was defined as:

Y_ijk_ = μ + T_i_ + P_j_ + (T × P)_ij_ + Cow_k_ + ε_ijk_,

where Y_ijk_ is the dependent variable, μ is the overall mean, T_i_ is the fixed effect of dietary treatment, P_j_ is the fixed effect of experimental period, (T × P)_ij_ is their interaction Cow_k_, is the random effect of cow, and ε_ijk_ is the residual error.

When repeated measurements over time were present, period-specific repeated measures were modeled within cow using an appropriate covariance structure selected based on model convergence and goodness of fit.

#### 2.9.2. Alpha and Beta Diversity Analysis

Alpha diversity indices of the rumen microbiota, including Observed features, Shannon diversity index, Faith’s phylogenetic diversity, and Pielou’s evenness, were calculated in QIIME 2 based on rarefied ASV tables. Differences in alpha diversity metrics were evaluated using linear mixed-effects models with dietary treatment, experimental period, and their interaction as fixed effects, and cow included as a random effect.

Beta diversity was assessed using Bray–Curtis dissimilarities and weighted UniFrac distances, calculated in QIIME 2 from rarefied ASV tables. Differences in microbial community structure were tested using permutational multivariate analysis of variance (PERMANOVA) with dietary treatment, experimental period, and their interaction included as explanatory variables. Statistical significance was assessed using 999 permutations. Community-level patterns were visualized using principal coordinates analysis (PCoA).

#### 2.9.3. qPCR Data Analysis

qPCR-derived bacterial abundance data were log10-transformed prior to statistical analysis to meet assumptions of normality and homoscedasticity. Pairwise comparisons between dietary treatments within each experimental period were performed using the Sidak adjustment for multiple comparisons.

#### 2.9.4. Environmental Fitting (Envfit) and Correlation-Based Integration Analysis

To identify associations between microbial community structure and ruminal, metabolic, and production-related variables, the envfit function was applied to PCoA ordinations using 999 permutations. Continuous variables, including ruminal pH, volatile fatty acids, nitrogen fractions, and biogenic amines, were fitted as vectors. Variables with *p* < 0.05 were considered significantly associated with microbial beta diversity.

To characterize dominant rumen taxa, a heatmap of the top 20 bacterial genera was generated based on mean relative abundances (log_10_-transformed after addition of a pseudo count, 1 × 10^−6^). Associations between genus-level relative abundance and host-related parameters were evaluated using Spearman’s rank correlations, including fermentation parameters, nitrogen fractions, biogenic amines, milk production traits, and digestibility. Significant correlations (*p* < 0.05) were visualized in a correlation heatmap.

## 3. Results

### 3.1. Sequencing Output and Data Quality

Quality-filtered 16S rRNA gene sequencing data comprised 45 rumen samples. The median sequencing depth was 17,961 reads per sample, with sequencing depth ranging from 8761 to 29,473 reads. To standardize sampling effort across samples, the feature table was rarefied to a depth of 17,000 sequences per sample prior to downstream analyses. This rarefaction depth retained the majority of samples while ensuring sufficient coverage for robust estimation of alpha and beta diversity metrics.

### 3.2. Alpha Diversity of the Rumen Microbiota

**Figure 1:**
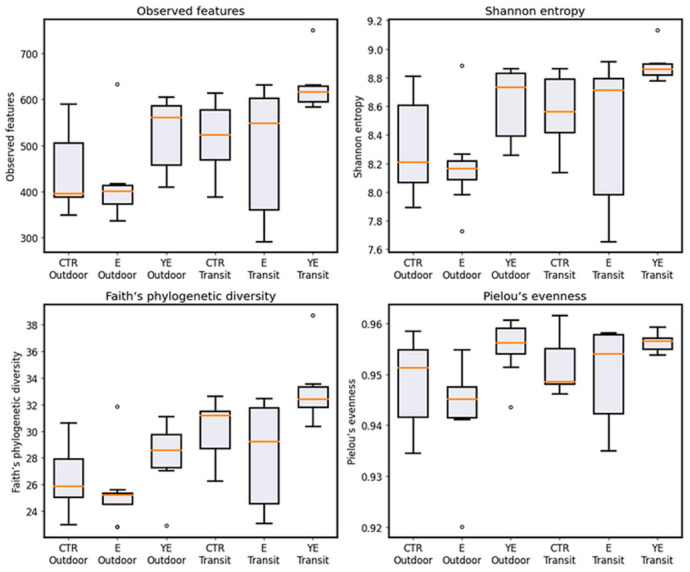
Alpha diversity of the rumen microbiota across dietary groups (CTR, E, and YE) and experimental periods (Outdoor and Transit).

Alpha diversity indices are presented in Figure 1. Pielou’s evenness was significantly affected by experimental period (*p* = 0.024), indicating differences between the OD and transit T periods, whereas the main effect of dietary treatment did not reach statistical significance, although a tendency toward group-related differences was observed (*p* = 0.086). No significant treatment × period interaction was detected (*p* = 0.215).

In contrast, richness and diversity metrics, including observed features, Faith’s phylogenetic diversity, and Shannon entropy, were significantly influenced by both dietary treatment (*p* = 0.007) and experimental period (*p* < 0.001), with no significant treatment × period interactions (*p* = 0.144). Across all treatments, these indices increased from the outdoor to the transit period. Moreover, values were consistently higher in the YE group compared with CTR and E, whereas no consistent differences were observed between group CTR and group E.

### 3.3. Beta Diversity of the Rumen Microbiota

**Figure 2:**
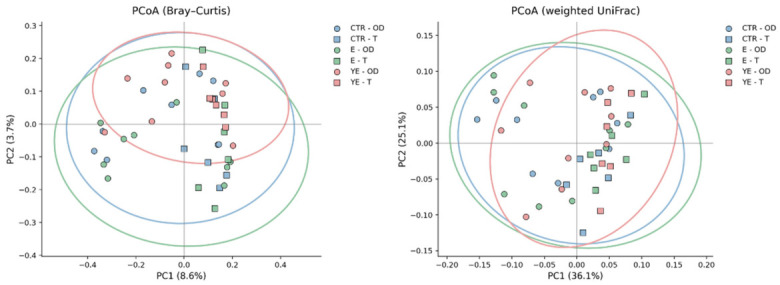
PCoA of rumen microbial community structure based on Bray–Curtis dissimilarities and weighted UniFrac distances across dietary treatments and experimental periods. Each point represents an individual sample; colors indicate dietary groups and shapes indicate experimental periods (outdoor vs. indoor). Ellipses represent 95% confidence intervals for each dietary group.

Beta diversity patterns are shown in Figure 2. PERMANOVA indicated that experimental period exerted a stronger influence on microbial community structure than dietary treatment, whereas the treatment × period interaction was not a major source of variation.

### 3.4. Relative Abundance of the Top 20 Bacterial Genus

**Figure 3:**
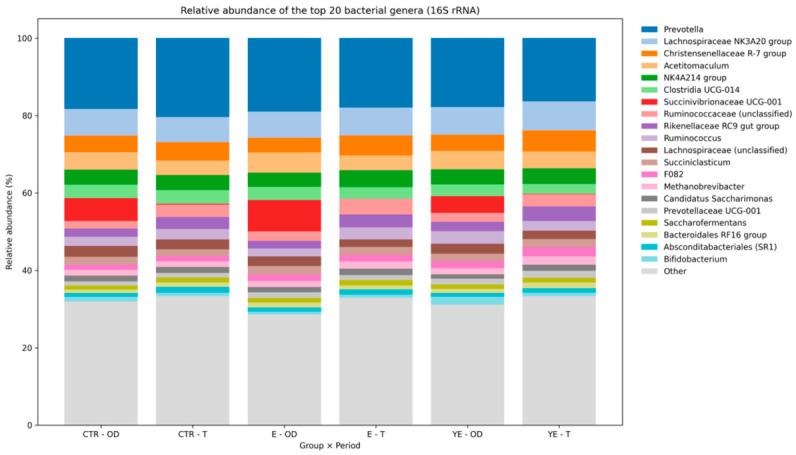
Relative abundance of the top 20 bacterial genera in the rumen microbiota across dietary treatments and experimental periods.

Across the 45 rumen samples, a total of 241 bacterial genera were identified at the genus level (Figure 3). The 20 most abundant genera were selected for further statistical evaluation. These taxa collectively accounted for 62.07 (3.15)% of total relative abundance, with individual samples ranging from 56.54% to 70.62%, indicating that the majority of the rumen bacterial community was represented by a limited number of dominant genera.

The rumen microbiota was numerically dominated by *Prevotella* spp. (18.36%), followed by *Lachnospiraceae* NK3A20 group (6.98%), *Christensenellaceae* R-7 group (4.50%), *Acetitomaculum* spp. (4.49%), *Lachnospiraceae* NK4A214 group (3.92%), and *Succinivibrionaceae* UCG-001 (3.73%). The remaining genera in Top 20 individually contributed between 0.43% and 2.67% of the total community. Together, these findings indicate a community structure primarily dominated by members of the phyla *Bacteroidota* and *Firmicutes*, with lower contributions from *Proteobacteria* and methanogenic archaea.

A comparison of the Top 20 genera between dietary treatments (CTR, E, and YE) revealed no significant differences. A tendency toward treatment-related variation was observed for *Ruminococcus* spp., in the OD period (*p* = 0.058), where mean relative abundance ranged from 2.05% (group E) to 3.20% (group YE). For all remaining genera, absolute differences within each group and period were small, generally below 1–2 percentage points, indicating limited dietary treatment influence on dominant rumen taxa.

In contrast, period-related comparisons within treatment groups revealed more pronounced shifts. Notably, *Ruminococcus* spp. increased within the E group from 2.05% (OD) to 3.15% (T) (*p* = 0.020). Additional genera exhibited tendencies toward OD–T variation within specific treatments. In group E, *Succinivibrionaceae* UCG-001 (*p* = 0.063) and *Christensenellaceae* R-7 group (*p* = 0.057) showed near-significant shifts between periods. Within the YE group, a tendency was observed for *Christensenellaceae* R-7 group (*p* = 0.095). In the CTR group, period-related trends were detected for *Rikenellaceae* RC9 group (*p* = 0.053) and *Ruminococcaceae* UCG-001 (*p* = 0.076). Although these effects did not reach statistical significance, they consistently indicated greater sensitivity to sampling period than to dietary treatment.

### 3.5. Effect of Dietary Treatment and Experimental Period on Rumen Bacterial Genera

Dietary treatment significantly affected several rumen bacterial genera (Table 2). Based on the LMM model, significant group effects (*p*_group_ < 0.05) were detected for *Ruminococcus* spp., *Prevotellaceae* UCG-003, uncultured *Bacteroidales*, *Blautia* spp., *WPS-2*, *Erysipelotrichaceae* UCG-009, and *Sharpea* spp. Cow groups received supplementation modified the abundance of specific bacterial taxa, with increased values of *Ruminococcus* spp. and *Prevotellaceae* UCG-003 in supplemented groups, whereas *Blautia* spp., *WPS-2*, and *Erysipelotrichaceae* UCG-009 were reduced compared with group CTR. Several additional genera showed trend-level effects (0.05 < *p* ≤ 0.10), indicating moderate diet-associated shifts in microbial structure.

When analyzed separately by period using one-way ANOVA, dietary group differences were primarily observed during the OD period, while no significant or trend-level differences were detected during the T period. During period OD, *Blautia* spp. was lower in E compared with group CTR by 0.151% (*p* < 0.01), *WPS-2* was reduced in group YE as compared to group CTR by 0.147%, and *Erysipelotrichaceae* UCG-009 was lower in group E compared with group CTR (by 0.103%). Trend-level differences during OD were observed for *Ruminococcus* spp., *Sharpea* spp., uncultured *Bacteroidales*, and *Prevotellaceae* UCG-003.

### 3.6. Total Bacterial Abundance Assessed by qPCR

Total rumen bacterial abundance, assessed by quantitative real-time PCR and expressed as log_10_-transformed 16S rRNA gene copy numbers per ng of genomic DNA, was significantly affected by experimental period (*p* < 0.001) (Table 3). In addition, a significant dietary treatment × period interaction was detected (*p* = 0.013), whereas the main effect of dietary treatment was not statistically significant (*p* = 0.298).

### 3.7. Rumen Fermentation Results

Ruminal pH was significantly affected by dietary treatment (*p* = 0.004) and experimental period (*p* < 0.001), with no significant treatment × period interaction detected (Table 4). Across treatments, pH was lower during the transition period compared with the outdoor period (*p* = 0.027) and was higher in the YE group compared with the E group (*p* = 0.013).

VFA concentration differed among dietary treatments (*p* = 0.025), with higher values observed in group E compared with group YE (*p* = 0.006), whereas no period or interaction effects were detected. Propionate concentration was influenced by both dietary treatment (*p* = 0.009) and experimental period (*p* < 0.001). The acetate-to-propionate (A:P) ratio was significantly affected by experimental period (*p* < 0.001), showing higher values during the transition period (T). Valerate concentration was also influenced by the experimental period (*p* = 0.018), with higher concentrations observed during the outdoor period (OD). Isobutyrate showed a treatment effect (*p* = 0.029), although pairwise comparisons did not reveal significant differences between dietary groups.

Total rumen nitrogen concentration exhibited a significant treatment × period interaction (*p* = 0.002) and was additionally affected by experimental period (*p* = 0.036). NH_3_–N concentration was influenced by experimental period (*p* = 0.013), with higher values observed during the outdoor period.

All other measured VFAs were not significantly affected by dietary treatment, experimental period, or their interaction (*p* > 0.05).

### 3.8. Biogenic Amines in Rumen Fluid Results

Results of rumen biogenic amine concentrations are presented in Table 5. Putrescine concentration was significantly affected by dietary treatment (*p* = 0.001), experimental period (*p* < 0.001), and their interaction (*p* = 0.001). During the outdoor period, putrescine concentration was higher in group E compared with both CTR (*p* = 0.004) and YE (*p* = 0.002), whereas no significant differences among dietary treatments were detected during the transition period. Across treatments, putrescine concentrations were lower during the transition period compared with the outdoor period. No significant effects of dietary treatment, experimental period, or their interaction were observed for spermidine (*p* > 0.05).

Cadaverine concentrations were characterized by a high proportion of zero values and substantial variance heterogeneity; therefore, results are reported descriptively without inferential statistical analysis. Histamine, tyramine, and spermine concentrations were predominantly zero across samples and were not subjected to formal statistical analysis. Tryptamine and phenylethylamine were not detected in rumen fluids.

### 3.9. Milk Yield and Composition Results

Milk yield was significantly affected by dietary treatment (*p* = 0.001), experimental period (*p* = 0.001), and their interaction (*p* = 0.001) (Table 6). During the OD period, milk yield was similar across groups (31–32 kg/d). In contrast, during the T period, cows in the YE group maintained a higher milk yield (30.48 kg/d) compared with the CTR (25.78 kg/d) and E (26.95 kg/d) groups.

Energy-corrected milk (ECM) yield was not affected by dietary treatment or treatment × period interaction (*p* > 0.05), but was influenced by experimental period (*p* = 0.006). Across all groups, ECM decreased during the transition period compared with the outdoor period.

Milk fat, protein, and lactose percentages were not significantly affected by dietary treatment, experimental period, or their interaction (*p* > 0.05). A tendency toward a treatment × period interaction was observed for milk fat (*p* = 0.075), and milk protein showed a tendency for a period effect (*p* = 0.058); however, these did not reach statistical significance.

### 3.10. Physical Assessment of Fecal Residues

The physical breakdown of feed, expressed as *WFF*, was not significantly affected by dietary treatment (*p* = 0.331), experimental period (*p* = 0.794), or their interaction (*p* = 0.306). *WFF* values were consistent across groups, ranging from approximately 70% to 77%. These results indicate that the physical comminution and washout rate of fecal particles remained stable regardless of the diet, suggesting that the fundamental rumen mechanical processing was maintained.

### 3.11. Integrative Association Analysis Results Between Rumen Microbiota and Host-Related Parameters

**Figure 4:**
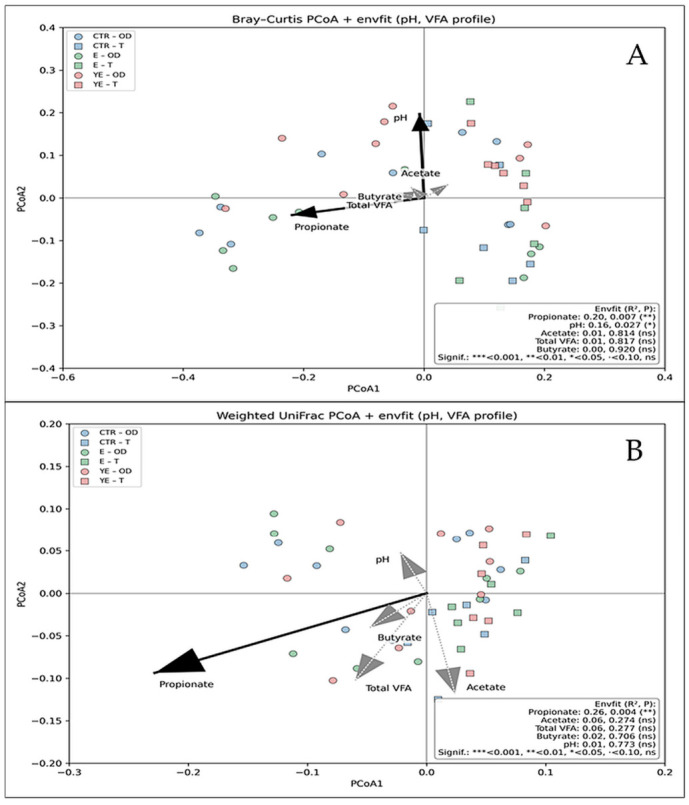
Bray–Curtis (**A**) and Weighted UniFrac (**B**) principal coordinates analysis (PCoA) of rumen microbial community composition with environmental fitting (envfit) of ruminal pH and major volatile fatty acids (VFA). Arrows indicate the direction and strength of associations between fermentation parameters and microbial beta diversity. Solid black vectors denote statistically significant associations (*p* < 0.05), whereas gray dashed vectors represent non-significant relationships. Points are colored by dietary treatment and shaped by an experimental period.

To evaluate associations between rumen fermentation characteristics and microbial community structure, envfit analysis was applied to PCoA ordinations based on Bray–Curtis and weighted UniFrac distances (Figure 4).

Based on Bray–Curtis dissimilarities, microbial community composition was significantly associated with propionate concentration (R^2^ = 0.20, *p* = 0.007) and ruminal pH (R^2^ = 0.16, *p* = 0.027). Acetate, total VFA, and butyrate were not significantly associated with Bray–Curtis ordination (*p* > 0.05). When phylogenetically weighted beta diversity was assessed using weighted UniFrac distances, propionate concentration remained significantly associated with microbial community structure (R^2^ = 0.26, *p* = 0.004), whereas no significant associations were detected for ruminal pH or other VFA parameters (*p* > 0.05).

**Figure 5:**
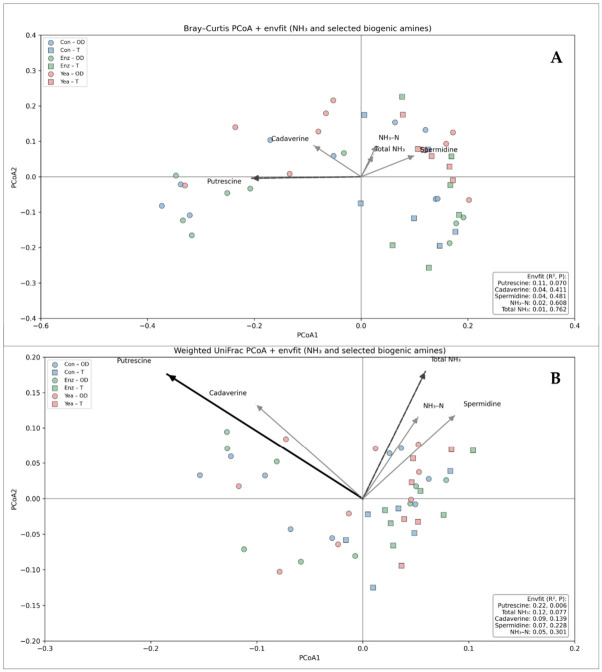
Bray–Curtis (**A**) and Weighted UniFrac (**B**) principal coordinates analysis (PCoA) of rumen microbial communities with environmental fitting of ammonia-related parameters and selected biogenic amines. PCoA based on Bray–Curtis dissimilarities illustrating variation in rumen microbial community composition across treatment groups and experimental periods. PCoA based on weighted UniFrac distances showing phylogenetically weighted differences in rumen microbial community structure across treatment groups and experimental periods. Envfit vectors represent significant or near-significant associations identified by envfit analysis for total NH_3_, NH_3_-N and selected biogenic amines (putrescine, cadaverine, and spermidine). Solid black vectors denote statistically significant associations (*p* < 0.05), dashed black vectors indicate trend-level associations (0.05 ≤ *p* < 0.10), whereas gray vectors represent non-significant relationships. Points are colored by dietary treatment and shaped by experimental period.

To examine associations between nitrogen metabolism and microbial community structure, envfit analysis was performed using total rumen nitrogen, NH_3_-N, and selected biogenic amines (Figure 5).

According to Bray–Curtis dissimilarities, a trend-level association was observed between microbial community composition and putrescine concentration (R^2^ = 0.11, *p* = 0.070). No significant associations were detected for total nitrogen, NH_3_-N, cadaverine, or spermidine (*p* > 0.05). In contrast, weighted UniFrac-based analysis revealed a significant association between microbial community structure and putrescine concentration (R^2^ = 0.22, *p* = 0.006). Total nitrogen showed a marginal association (R^2^ = 0.12, *p* = 0.077), whereas NH_3_-N, cadaverine, and spermidine were not significantly associated with phylogenetically weighted beta diversity (*p* > 0.05).

To assess potential associations between rumen microbial community structure and productive performance, envfit analyses were applied. None of the evaluated milk production traits showed significant associations with microbial beta diversity based on either Bray–Curtis dissimilarities or weighted UniFrac distances.

**Figure 6:**
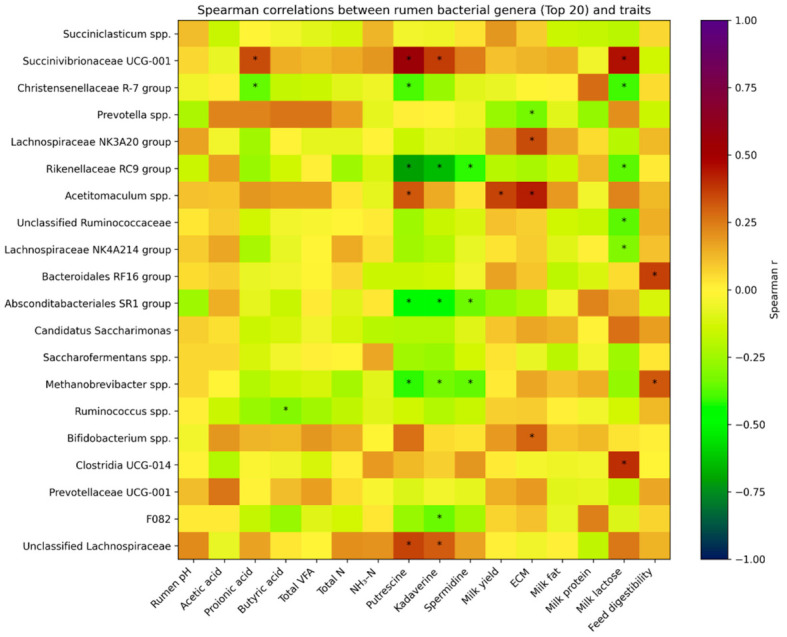
Spearman correlation heatmap between rumen bacterial genus and ruminal, metabolic, and production traits. Feed digestibility was estimated as the washable fecal fraction (*WFF*). Asterisks (*) indicate statistically significant Spearman correlations (*p* < 0.05).

To further explore associations between bacterial taxa and host-related parameters, a correlation heatmap was constructed based on Spearman’s rank correlation coefficients between the relative abundance of the top 20 bacterial genus and rumen fermentation parameters, nitrogen fractions, biogenic amines, and milk production traits. Statistically significant correlations (*p* < 0.05) are indicated in the heatmap (Figure 6).

The strongest correlations were observed with biogenic amines. *Rikenellaceae* RC9 group exhibited a strong negative association with putrescine (r = −0.71, *p* < 0.001) and cadaverine (r = −0.65, *p* < 0.001), and was additionally negatively correlated with spermidine (r = −0.41, *p* = 0.005). In contrast, *Succinivibrionaceae* spp. UCG-001 showed positive correlations with putrescine (r = 0.56, *p* < 0.001) and cadaverine (r = 0.37, *p* = 0.012). Similarly, *Absconditabacteriales* SR1 group was negatively associated with cadaverine (r = −0.48, *p* < 0.001) and putrescine (r = −0.46, *p* = 0.001). Additional negative associations with biogenic amines were observed for *Methanobrevibacter* spp. (putrescine: r = −0.42, *p* = 0.004; spermidine: r = −0.37, *p* = 0.013) and *Christensenellaceae* R-7 group (putrescine: r = −0.39, *p* = 0.008). These results indicate a consistent linkage between specific genera and amine metabolism in the rumen ecosystem.

Associations with fermentation and production parameters were comparatively weaker and observed only for specific taxa. *Christensenellaceae* R-7 group was negatively correlated with propionic acid (r = −0.36, *p* = 0.016), whereas *Bacteroidales* RF16 group showed a positive association with feed digestibility (r = 0.36, *p* = 0.015). Regarding production traits, *Acetitomaculum* spp. was positively associated with ECM (r = 0.43, *p* = 0.004) and milk yield (r = 0.36, *p* = 0.016), while *Succinivibrionaceae* UCG-001 was positively correlated with milk lactose (r = 0.45, *p* = 0.002). Conversely, *Christensenellaceae* R-7 group, *Rikenellaceae* RC9 group, and unclassified *Ruminococcaceae* showed negative correlations with milk lactose (r ranging from −0.41 to −0.37, *p* < 0.02). Overall, correlation patterns were more pronounced for ruminal metabolic parameters, particularly biogenic amines, than for milk production traits, indicating tighter functional links between microbial composition and rumen metabolic activity than with downstream production outcomes.

## 4. Discussion

The present study evaluated the effects of dietary supplementation with exogenous enzymes alone or in combination with live yeast on rumen microbial community structure, fermentation characteristics, and productive performance in dairy cows during the transition from outdoor grazing to indoor housing. Rumen microbial composition was influenced more by dietary changes associated with the experimental period than by supplementation, whereas supplementation primarily affected specific bacterial groups and functional parameters. These results indicate that changes in microbial composition were not always directly linked to changes in fermentation or production outcomes, and therefore microbiota data should be interpreted together with metabolic and fermentation results.

### 4.1. Effects of Supplementation on Microbial Community Structure and Abundance Dynamics

Beta diversity analyses based on both Bray–Curtis dissimilarities and weighted UniFrac distances demonstrated a statistically significant separation between the outdoor (OD) and transit (T) periods (*p* = 0.047), whereas differences among dietary treatments within the same period did not show consistent or statistically significant separation. This indicates that substantial changes in rumen microbial diversity require major shifts in feeding and housing conditions, whereas supplementation with enzymes or enzymes combined with live yeast alone was not sufficient to induce comparable changes at the alpha or beta diversity level. It should be noted that the relatively small number of animals used for rumen microbiota analysis may have limited the ability to detect subtle differences between dietary treatments, particularly in the context of inherent inter-animal variability. However, the selected cows were highly comparable, as they were chosen according to the analog principle and did not differ significantly in baseline characteristics, which helped reduce variability within the dataset.

In this context, the observed clear separation between experimental periods, but not between dietary treatments, suggests that major shifts in feeding and management conditions exert a stronger influence on rumen microbial community structure than supplementation alone. Similar patterns have been reported in studies showing that transitions between grazing and indoor feeding systems strongly influence rumen microbial dynamics [4,28]. Nevertheless, alternative experimental designs, including larger sample sizes, different feeding strategies, or variation in breed and physiological status, may allow detection of more subtle treatment-related effects that could not be captured in the present study.

Comparable temporal patterns have been reported in studies examining transitions between pasture-based and indoor TMR feeding systems. Changes in forage composition, feeding schedule, and nutrient supply were identified as major factors shaping rumen microbiota dynamics. For example, Schären et al. [4] showed that shifts between grazing and TMR feeding led to gradual changes in bacterial diversity and community structure, reflecting microbial adaptation to new substrate conditions. Similarly, Noel and Moloney [28] reported seasonal changes in rumen bacterial communities in grazing cows. Additionally, studies on rumen microbial colonization have shown that microbial communities gradually change when the composition of the feed changes, reflecting adaptation of bacteria to new substrates over time [29]. Together, these studies show that time and management changes act as strong factors naturally shaping rumen microbiota composition, even without dietary supplementation.

Alpha diversity results showed a similar pattern. Microbial richness and phylogenetic diversity increased during the transition period (*p* < 0.001), meaning that more different types of bacteria were detected after cows moved indoors. Dietary treatment also affected several alpha diversity measures (*p* < 0.001), with the highest values observed in the YE group. Although alpha diversity increased during the transition period and in the YE group, these changes were not consistently associated with improvements in fermentation parameters or production traits. This supports findings from other studies showing that alpha diversity alone is not a reliable indicator of rumen efficiency or animal performance. Instead, functional outcomes appear to depend more on the activity and abundance of specific microbial groups than on overall community diversity [30].

Genus-level analysis further confirmed that dietary effects were selective rather than community-wide. The linear mixed model analysis demonstrated that several genera responded significantly to dietary supplementation, indicating that these shifts were associated with the applied feed additives rather than solely with the transition period. In particular, *Prevotellaceae* UCG-003 increased in the supplemented groups, which is consistent with previous findings showing that yeast-based direct-fed microbials can modulate this taxon in the rumen [31]. Similarly, *Blautia* spp. has been reported to respond to microbial supplementation, further supporting the interpretation that this genus is sensitive to additive-dependent metabolic changes rather than only to basal diet composition. The increase in *Ruminococcus* spp. in supplemented groups may reflect enhanced substrate availability, as members of this genus are key degraders of structural and non-structural carbohydrates and play a central role in ruminal fermentation [32]. Moreover, the observed changes in *Sharpea* spp. can be explained by its role in lactate and short-chain fatty acid metabolism and its reported associations with productive performance and rumen fermentation dynamics [33].

Quantitative PCR analysis showed that total bacterial abundance was strongly influenced by the experimental period (*p* = 0.001), indicating an overall reduction during the transition from outdoor to indoor conditions. The main effect of dietary treatment was not significant (*p* = 0.298). However, a significant group x period interaction (*p* = 0.013) was detected, suggesting that the response to supplementation depended on the experimental period. During the outdoor period, total bacterial numbers were similar across groups. In contrast, during the transition period, the YE group showed a greater decrease than the other treatments. In line with other studies [13,34], this suggests that the effect of yeast supplementation depended on the feeding period. Instead of showing a consistent effect across periods, yeast supplementation influenced bacterial abundance specifically during the dietary transition.

### 4.2. Effects of Supplementation on Rumen Fermentation and Metabolic Parameters

Dietary supplementation significantly affected several rumen fermentation parameters. Total VFA concentration differed among groups (*p* = 0.025), with higher values observed in group E compared with YE, while propionate concentration was influenced by both dietary treatment (*p* = 0.009) and experimental period (*p* < 0.001). Ruminal pH was also affected by treatment (*p* = 0.004), with higher values in the YE group compared with E. In addition, total rumen nitrogen exhibited a significant treatment × period interaction (*p* = 0.002), and putrescine concentration was strongly influenced by treatment, period, and their interaction (*p* = 0.001). These findings are consistent with previous studies showing that exogenous fibrolytic enzymes can alter rumen fermentation patterns by improving plant fiber degradation and increasing the availability of fermentable substrates for rumen microbes [8,10,11]. In this context, the observed increase in propionate suggests a shift in carbohydrate fermentation toward more glucogenic pathways. This interpretation is supported by integrative multivariate analyses, which showed that propionate was the fermentation parameter most strongly associated with microbial community structure in both Bray–Curtis and weighted UniFrac analyses. Together, these results indicate that changes in fermentation profiles were linked to coordinated responses among metabolically related microbial groups rather than occurring independently [35,36,37].

Putrescine was the only biogenic amine that showed clear treatment- and period-dependent differences in our study. Its concentration was significantly influenced by supplementation, experimental period, and their interaction, indicating that its response depended on feeding conditions. Putrescine is formed through microbial amino acid degradation and indicates nitrogen metabolism in the rumen [38,39]. The higher concentrations observed in the only enzyme supplemented group during the outdoor period suggest that exogenous enzymes may have enhanced substrate availability not only for carbohydrate fermentation but also for microbial protein and amino acid turnover. At the same time, the strong decline in putrescine during the transition period across all groups highlights the dominant effect of feeding changes on nitrogen-related pathways [40,41]. Integrative analyses further showed that putrescine was associated with microbial community structure, particularly in beta diversity analyses. In contrast, other nitrogen fractions and biogenic amines did not show consistent relationships with microbial structure. This selectivity suggests that supplementation influenced specific nitrogen metabolic pathways rather than causing a general increase in protein breakdown [38,42]. As biogenic amines are continuously produced and degraded in the rumen, their concentrations reflect ongoing microbial activity. When considered together with volatile fatty acids, biogenic amines (particularly putrescine) provide additional insight into functional changes within the overall rumen ecosystem.

### 4.3. Effect of Supplementation on Milk Production and Composition

The significant group × period interaction for milk yield indicates that the diet treatments differentially influenced cows’ ability to maintain production during the transit period. This effect may be attributed to differential modulation of rumen fermentation processes by the dietary treatments, which may have influenced nutrient availability and, consequently, milk production responses. While milk yield declined markedly in the CTR and E groups, cows receiving the YE treatment showed a much smaller reduction, suggesting improved resilience to the physiological or environmental challenges associated with the transit period. This finding implies that the YE treatment may support sustained lactational performance under changing management or environmental conditions.

In contrast, ECM was primarily affected by period rather than treatment, indicating that the overall energy output in milk declined during the transit period regardless of diet. This suggests that although YE cows maintained higher milk volume, changes in milk composition, particularly milk fat, partly offset the energy value of milk. The lack of a treatment effect on ECM highlights that improvements in raw milk yield do not necessarily translate into proportional increases in energy-corrected production.

A large analysis of publications showed that yeast culture supplementation significantly increases milk yield across many trials in Holstein cows. This suggests that products based on Saccharomyces cerevisiae can support lactational performance, especially when strategically dosed and applied in specific lactation stages [43]. Several individual trials and systematic reviews also report numerical or significant increases in milk yield with yeast products, though the magnitude varies with dose and diet composition [9,44].

These effects are generally attributed to improved rumen fermentation efficiency, including stabilization of ruminal pH, stimulation of fibrolytic microbial activity, and enhanced microbial protein synthesis, which together support greater nutrient availability for milk production. This is consistent with our findings, where the YE group maintained a higher ruminal pH, indicating a more stable rumen environment under changing feeding conditions. Notably, milk yield was similar across groups during the grazing period, whereas clear differences emerged during the transition period, with the YE group showing a smaller decline, suggesting that improved rumen stability may have supported more consistent milk production during the transition period.

Some studies report increased milk fat with yeast culture supplementation, but responses are variable and sometimes non-significant—similar to our findings [44]. Many trials demonstrate improved protein content when yeast products enhance rumen microbial protein synthesis, though not all studies reach statistical significance [43]. Lactose production is tightly regulated physiologically and less influenced by diet compared with protein and fat, which fits our results and broader dairy nutrition research [9].

## 5. Conclusions

This study showed that dietary supplementation with exogenous fibrolytic enzymes, alone or in combination with live yeast, resulted in selective changes in specific rumen bacterial genera and was associated with alterations in fermentation patterns and nitrogen-related metabolism, particularly in propionate and putrescine concentrations. At the same time, the transition from outdoor grazing to indoor feeding was a major factor influencing rumen microbial dynamics. The combination of enzymes and live yeast did not produce clear synergistic effects on microbial composition or fermentation parameters. However, cows receiving the combined supplementation maintained milk yield more effectively during the transition period compared with the control and enzyme-only groups. Overall, the results partially support our hypothesis and indicate that dietary supplementation can modulate specific rumen bacterial genera and metabolic responses during the transition from outdoor grazing to indoor feeding, thereby contributing to improved production stability.

## Figures and Tables

**Table 1:** Ingredient and chemical composition of experimental diets during grazing (Outdoor, OD) and indoor (Transit, T) periods.

**Ingredient Composition (% of Diet)**		
**Parameter**	**OD**	**T**
Grass silage ^1^	26.0	42.8
Maize silage ^1^	9.0	12.3
Wheat straw ^1^	3.0	5.1
Crushed maize grain ^1^	5.0	9.1
Concentrate mixture ^1,2^	29.0	30.7
Pasture herbage ^3^	28.0	0.0
**Chemical Composition**		
**Parameter**	**OD**	**T**
Dry matter (DM, %)	40.5	44.1
Organic matter (OM, % DM)	71.1	74.6
Crude protein (CP, % DM)	15.08	13.6
Neutral detergent fiber (NDF, % DM)	43.11	37.1
Acid detergent fiber (ADF, % DM)	27.46	22.2

^1^ Included in the total mixed ration (TMR). ^2^ Concentrate mixture consisted of cereal grains (wheat, triticale, oats), pea grain, field beans, rapeseed meal, soybean meal, sodium bicarbonate, limestone, sodium chloride, and vitamin–mineral premix. ^3^ Botanical composition of grass silage and of pasture herbage: timothy (*Phleum pratense*), red clover (*Trifolium pratense*), white clover (*Trifolium repens*), and lucerne (*Medicago sativa*).

**Table 2:** Estimated marginal means (SD) of rumen bacterial genera relative abundance (%) according to dietary treatment and experimental period, analyzed using linear mixed-effects models.

Treatment Group	CTR		E		YE		*p* Value		
Genus/Period	OD	T	OD	T	OD	T	*p_g_*_roup_	*p*_period_	*p*_(g×p)_
*Blautia* spp.	0.237 (0.14)	0.286 (0.09)	0.086 (0.04)	0.297 (0.18)	0.294 (0.14)	0.277 (0.07)	0.001	0.744	0.151
*WPS-2*	0.163 (0.08)	0.164 (0.13)	0.057 (0.03)	0.065 (0.04)	0.016 (0.01)	0.117 (0.13)	0.002	0.656	0.243
*Ruminococcus* spp.	2.361 (0.91)	2.670 (0.41)	2.047 (0.56)	3.150 (0.45)	3.198 (0.60)	2.498 (0.67)	0.009	0.274	0.012
*Erysipelotrichaceae* UCG-009	0.136 (0.08)	0.184 (0.05)	0.033 (0.03)	0.101 (0.02)	0.108 (0.02)	0.202 (0.12)	0.010	0.244	0.895
*Sharpea* spp.	1.454 (0.43)	0.460 (0.55)	0.456 (0.41)	0.492 (0.82)	1.406 (1.07)	0.895 (0.64)	0.019	0.001	0.047
Uncultured *Bacteroidales*	0.109 (0.07)	0.193 (0.08)	0.189 (0.10)	0.215 (0.10)	0.154 (0.03)	0.253 (0.10)	0.041	0.180	0.643
*Eubacterium coprostanoligenes* group	0.438 (0.16)	0.417 (0.11)	0.627 (0.05)	0.651 (0.29)	0.704 (0.33)	0.715 (0.21)	0.062	0.985	0.927
*Prevotellaceae* UCG-003	0.643 (0.26)	0.908 (0.22)	0.569 (0.19)	1.049 (0.20)	0.809 (0.11)	1.012 (0.12)	0.046	0.025	0.196
*Lachnospiraceae* NK4A136 group	0.209 (0.06)	0.259 (0.07)	0.11 (0.05)	0.120 (0.05)	0.120 (0.02)	0.09 (0.02)	0.084	0.639	0.031
*Lachnospira* spp	0.389 (0.01)	0.364 (0.01)	0.253 (0.05)	0.276 (0.03)	0.277 (0.05)	0.232 (0.06)	0.086	0.956	0.490
*Moryella* spp	0.456 (0.03)	0.330 (0.02)	0.319 (0.04)	0.364 (0.09)	0.552 (0.31)	0.355 (0.07)	0.089	0.264	0.306
*Bacteroidales* RF16 group	0.898 (0.51)	1.191 (0.26)	1.320 (0.19)	1.064 (0.17)	1.057 (0.16)	1.478 (0.35)	0.098	0.100	0.175
*Fibrobacter* spp.	0.157 (0.08)	0.606 (0.15)	0.317 (0.15)	0.647 (0.12)	0.421 (0.18)	0.724 (0.31)	0.099	0.006	0.489
*F082*	1.325 (0.13)	1.390 (0.29)	1.619 (0.49)	1.693 (0.11)	1.795 (0.28)	2.381 (0.57)	0.099	0.954	0.569
*Lachnospiraceae* AC2044 group	0.178 (0.09)	0.261 (0.09)	0.090 (0.08)	0.358 (0.12)	0.197 (0.03)	0.267 (0.04)	0.100	0.632	0.120

Relative abundances are expressed as percentages (%) of the total bacterial community. Values are presented as mean ± (SD). *p*-values were obtained from linear mixed-effects models including dietary group, experimental period, and their interaction as fixed effects, with cow included as a random effect. Pairwise comparisons were adjusted using the Sidak correction.

**Table 3:** Effects of dietary treatments and experimental period on rumen total bacterial abundance assessed by qPCR.

Treatment Group	CTR		E		YE		*p* Value		
Genus/Period	OD	T	OD	T	OD	T	*p*_group_	*p_p_*_eriod_	*p*_(g×p)_
log_10_(copies/ng DNA)	5.43 (0.08)	5.36 (0.02)	5.42 (0.03)	5.38 (0.05)	5.40 (0.02)	5.29 (0.09)	0.298	0.001	0.013

Values are presented as mean ± (SD). Data are expressed as log10-transformed 16S rRNA gene copy numbers per ng of genomic DNA. OD—outdoor period; T—transit period. *p*_group_, *p*_period_, and *p*_(g×p)_ indicate fixed effects derived from the linear mixed-effects model. Pairwise comparisons were adjusted using the Sidak correction.

**Table 4:** Rumen fermentation parameters, volatile fatty acid concentrations, and nitrogen fractions in dairy cows across treatment groups and periods.

Treatment Group	CTR		E		YE		*p* Value		
Parameter/Period	OD	T	OD	T	OD	T	*p*_group_	*p*_period_	*p*_(g×p)_
Rumen pH	6.36 (0.17)	6.25 (0.13)	6.25 (0.08)	6.03 (0.12)	6.54 (0.08)	6.27 (0.19)	0.004	0.001	0.766
Total VFA	79.73 (12.76)	79.05 (12.92)	87.43 (12.26)	73.51 (5.86)	73.87 (8.41)	65.96 (15.83)	0.025	0.125	0.343
Acetate	47.74 (4.27)	50.2 (7.3)	52.68 (2.75)	48.0 (2.95)	45.51 (5.85)	43.29 (11.23)	0.062	0.442	0.384
Propionate	14.13 (5.67)	12.32 (2.88)	15.86 (2.9)	10.08 (0.92)	11.99 (1.15)	8.41 (1.29)	0.009	0.001	0.530
Butyrate	11.32 (2.17)	10.54 (2.17)	12.66 (1.58)	10.1 (2.11)	10.96 (2.46)	9.48 (3.23)	0.325	0.661	0.733
A:P ratio	3.79 (1.12)	4.36 (0.17)	3.42 (0.8)	4.82 (0.3)	3.87 (0.65)	5.17 (0.66)	0.725	0.001	0.362
Valerate	2.35 (0.71)	1.77 (0.22)	2.1 (0.11)	1.57 (0.09)	1.91 (0.15)	1.38 (0.13)	0.073	0.018	0.680
Caproate	1.017 (0.57)	1.06 (0.09)	1.1 (0.28)	1.04 (0.02)	1.03 (0.19)	0.93 (0.09)	0.507	0.655	0.668
Isobutyrate	1.16 (0.13)	1.2 (0.18)	1.1 (0.02)	0.99 (0.13)	0.98 (0.15)	0.94 (0.06)	0.029	0.953	0.756
n-Heptanoate	0.49 (0.01)	0.49 (0.01)	0.49 (0.02)	0.5 (0.01)	0.49 (0.01)	0.48 (0.01)	0.385	0.968	0.201
3-Methylvalerate	1.52 (0.25)	1.46 (0.14)	1.35 (0.13)	1.23 (0.11)	1.01 (0.08)	1.04 (0.2)	1.0	1.0	1.0
Total N	98.16 (10.27)	66.85 (8.23)	96.27 (22.99)	94.27 (13.3)	89.76 (9.57)	89.71 (25.19)	0.752	0.036	0.002
NH_3_–N	18.79 (0.59)	14.05 (3.13)	17.61 (3.6)	19.18 (2.66)	20.03 (1.18)	18.85 (2.46)	0.237	0.013	0.144

Abbreviations: VFA—volatile fatty acids (mmol/L); A:P ratio—acetate-to-propionate ratio; Total N—total rumen nitrogen (mg/dL); NH_3_–N—rumen ammonia nitrogen (mg/dL). Values are presented as mean (SD). *p*_group_, *p*_period_, and *p*_(g×p)_ indicate fixed effects derived from the linear mixed-effects model. Values are presented as mean ± (SD). Pairwise comparisons were adjusted using the Sidak correction.

**Table 5:** Rumen biogenic amine concentrations in dairy cows across treatment groups and experimental periods.

Treatment Group	CTR		E		YE		*p* Value		
Parameter/Period	OD	T	OD	T	OD	T	*p*_group_	*p*_period_	*p*_(g×p)_
Putrescine	24.04 (3.38)	3.11 (0.46)	29.54 (3.79)	4.83 (0.94)	27.04 (12.24)	4.88 (1.67)	0.001	0.001	0.001
Cadaverine	28.62 (10.16)	4.98 (0.91)	21.14 (10.17)	6.56 (2.95)	23.34 (6.86)	7.33 (4.95)	na *	na *	na *
Histamine	0.97 (0.94)	0.0	0.11 (0.18)	0.0	0.45 (0.78)	0.17 (0.3)	na **	na **	na **
Tyramine	0.74 (1.28)	0.0	0.0	0.0	0.54 (0.93)	0.0	na **	na **	na **
Spermidine	6.24 (1.86)	3.06 (1.32)	4.96 (3.04)	3.1 (1.06)	7.12 (2.91)	4.42 (2.99)	0.186	0.983	0.876
Spermine	0.75 (0.65)	0.0	0.19 (0.19)	0.7 (0.47)	0.36 (0.32)	1.06 (0.66)	na **	na **	na **

All biogenic amine concentrations are expressed as mg kg^−1^ of rumen content (mean (SD)). Tryptamine and Phenylethylamine were not detected in rumen samples. na * Cadaverine concentrations exhibited a high proportion of zero values and extreme variance heterogeneity; therefore, linear mixed-effects model assumptions were violated, and no inferential statistics were reported. na ** Histamine, Tyramine and Spermine concentrations were predominantly zero across samples, resulting in insufficient variability for linear mixed-effects model estimation; therefore, statistical inference for fixed effects was not performed. *p*_group_, *p*_period_, and *p*_(g×p)_ indicate fixed effects derived from the linear mixed-effects model. Pairwise comparisons were adjusted using the Sidak correction.

**Table 6:** Milk yield and milk composition parameters in dairy cows across treatment groups and periods.

Treatment Group	CTR		E		YE		*p* Value		
Parameter/Period	OD	T	OD	T	OD	T	*p*_group_	*p*_period_	*p*_(g×p)_
Milk yield (kg/d)	31.76 (4.02)	25.78 (5.66)	31.32 (2.58)	26.95 (3.07)	31.3 (6.96)	30.48 (6.52)	0.001	0.001	0.001
ECM (kg/d)	33.48 (4.01)	27.87 (6.08)	32.21 (2.37)	28.6 (4.81)	33.63 (8.03)	29.48 (3.67)	0.736	0.006	0.400
Milk fat (%)	4.58 (0.62)	4.63 (0.29)	4.34 (0.4)	4.6 (0.43)	4.69 (0.24)	3.81 (0.71)	0.596	0.681	0.075
Milk protein (%)	3.3 (0.13)	3.55 (0.09)	3.28 (0.29)	3.19 (0.22)	3.14 (0.26)	3.16 (0.17)	0.188	0.058	0.648
Milk lactose (%)	4.48 (0.98)	4.48 (0.08)	4.71 (0.35)	4.61 (0.08)	4.74 (0.09)	4.59 (0.19)	1.000	0.998	1.000

ECM—energy-corrected milk. Values are presented as mean ± (SD). *p*_group_, *p*_period_, and *p*_(g×p)_ indicate fixed effects derived from the linear mixed-effects model. Pairwise comparisons were adjusted using the Sidak correction.

## Data Availability

The original contributions presented in this study are included in the article. Further inquiries can be directed to the corresponding authors.

## References

[1] Perlman D., Martínez-Álvaro M., Moraïs S., Altshuler I., Hagen L.H., Jami E., Roehe R., Pope P.B., Mizrahi I. (2022). Concepts and Consequences of a Core Gut Microbiota for Animal Growth and Development. Annu. Rev. Anim. Biosci..

[2] Yang Z., Li X., Hao Y., Ren K., Liu S., Cao Z., Wang Y., Yang H., Li S., Wang W. (2025). Quantitative microbiome profiling reveals associations between ruminal microbiota shifts and accelerated milk production increase in dairy cows. Food Res. Int..

[3] Wallace R.J., Sasson G., Garnsworthy P.C., Tapio I., Gregson E., Bani P., Huhtanen P., Bayat A.R., Strozzi F., Biscarini F. (2019). A heritable subset of the core rumen microbiome dictates dairy cow productivity and emissions. Sci. Adv..

[4] Schären M., Kiri K., Riede S., Gardener M., Meyer U., Hummel J., Urich T., Breves G., Dänicke S. (2017). Alterations in the Rumen Liquid-, Particle- and Epithelium-Associated Microbiota of Dairy Cows during the Transition from a Silage- and Concentrate-Based Ration to Pasture in Spring. Front. Microbiol..

[5] Jami E., Mizrahi I. (2012). Composition and Similarity of Bovine Rumen Microbiota across Individual Animals. PLoS ONE.

[6] Henderson G., Cox F., Ganesh S., Jonker A., Young W., Janssen P.H., Global Rumen Census Collaborators (2015). Rumen microbial community composition varies with diet and host, but a core microbiome is found across a wide geographical range. Sci. Rep..

[7] Nakano M.S.H., Tohno M., Matoba K., Uegaki R., Ishizaki H. (2013). Variation of rumen bacterial diversity in steers after the beginning of grazing. Proceedings of the 22nd International Grassland Congress, Sydney, Australia, 15–19 September 2013.

[8] Beauchemin K.A., Colombatto D., Morgavi D.P., Yang W.Z. (2003). Use of Exogenous Fibrolytic Enzymes to Improve Feed Utilization by Ruminants. J. Anim. Sci..

[9] Arriola K.G., Oliveira A.S., Ma Z.X., Lean I.J., Giurcanu M.C., Adesogan A.T. (2017). A meta-analysis on the effect of dietary application of exogenous fibrolytic enzymes on the performance of dairy cows. J. Dairy Sci..

[10] Tirado-González D.N., Miranda-Romero L.A., Ruíz-Flores A., Medina-Cuéllar S.E., Ramírez-Valverde R., Tirado-Estrada G. (2018). Meta-analysis: Effects of exogenous fibrolytic enzymes in ruminant diets. J. Appl. Anim. Res..

[11] Kondratovich L.B., Sarturi J.O., Hoffmann C.A., Ballou M.A., Trojan S.J., Campanili P.R.B. (2019). Effects of dietary exogenous fibrolytic enzymes on ruminal fermentation characteristics of beef steers fed high- and low-quality growing diets. J. Anim. Sci..

[12] Baker L.M., Kraft J., Karnezos T.P., Greenwood S.L. (2022). Review: The effects of dietary yeast and yeast-derived extracts on rumen microbiota and their function. Anim. Feed Sci. Technol..

[13] Jiang Q., Sherlock D.N., Elolimy A.A., Yoon I., Loor J.J. (2024). Feeding a Saccharomyces cerevisiae fermentation product during a gut barrier challenge in lactating Holstein cows impacts the ruminal microbiota and metabolome. J. Dairy Sci..

[14] Cagle C.M., Fonseca M.A., Callaway T.R., Runyan C.A., Cravey M.D., Tedeschi L.O. (2020). Evaluation of the effects of live yeast on rumen parameters and in situ digestibility of dry matter and neutral detergent fiber in beef cattle fed growing and finishing diets. Appl. Anim. Sci..

[15] State Animal Breeding Supervision Service under the Ministry of Agriculture (2010). Procedure for Inspections of Persons Performing Productivity Control of Dairy Livestock.

[16] Bureenok S., Yuangklang C., Vasupen K., Schonewille J.T., Kawamoto Y. (2012). The effects of additives in Napier grass silages on chemical composition, feed intake, nutrient digestibility and rumen fermentation. Asian-Australas. J. Anim. Sci..

[17] Ben-Gigirey B., De Sousa J.M.V.B., Villa T.G., Barros-Velazquez J. (1999). Histamine and cadaverine production by bacteria isolated from fresh and frozen albacore (*Thunnus alalunga*). J. Food Prot..

[18] Bartkienė E., Juodeikienė G., Vidmantienė D., Viskelis P., Urbonavičienė D. (2011). Nutritional and quality aspects of wheat sourdough bread using *L. luteus* and *L. angustifolius* flours fermented by *Pediococcus acidilactici*. Int. J. Food Sci. Technol..

[19] Stern M.D., Endres M.I. (1991). Laboratory Manual: Research Techniques in Ruminant Physiology.

[20] Rius A.G., Kittelmann S., Macdonald K.A., Waghorn G.C., Janssen P.H., Sikkema E. (2012). Nitrogen metabolism and rumen microbial enumeration in lactating cows with divergent residual feed intake fed high-digestibility pasture. J. Dairy Sci..

[21] Huuki H., Ahvenjärvi S., Lidauer P., Popova M., Vilkki J., Vanhatalo A., Tapio I. (2022). Fresh rumen liquid inoculant enhances the rumen microbial community establishment in pre-weaned dairy calves. Front. Microbiol..

[22] Edwards J.E., Huws S.A., Kim E.J., Kingston-Smith A.H. (2007). Characterization of the dynamics of initial bacterial colonization of nonconserved forage in the bovine rumen. FEMS Microbiol. Ecol..

[23] Caporaso J.G., Lauber C.L., Walters W.A., Berg-Lyons D., Lozupone C.A., Turnbaugh P.J., Fierer N., Knight R. (2011). Global patterns of 16S rRNA diversity at a depth of millions of sequences per sample. Proc. Natl. Acad. Sci. USA.

[24] Bolyen E., Rideout J.R., Dillon M.R., Bokulich N.A., Abnet C.C., Al-Ghalith G.A., Alexander H., Alm E.J., Arumugam M., Asnicar F. (2019). Reproducible, interactive, scalable and extensible microbiome data science using QIIME 2. Nat. Biotechnol..

[25] Callahan B.J., McMurdie P.J., Rosen M.J., Han A.W., Johnson A.J.A., Holmes S.P. (2016). DADA2: High-resolution sample inference from Illumina amplicon data. Nat. Methods.

[26] Quast C., Pruesse E., Yilmaz P., Gerken J., Schweer T., Yarza P., Peplies J., Glöckner F.O. (2013). The SILVA ribosomal RNA gene database project: Improved data processing and web-based tools. Nucleic Acids Res..

[27] Hall M.B., D’Mello J.P.F. (2002). Determination of starch, protein, fat, fiber, and ash in animal feed; methods for estimating digestibility. Handbook of Animal Nutrition.

[28] Noel S.J., Attwood G.T., Rakonjac J., Moon C.D., Waghorn G.C., Janssen P.H. (2017). Seasonal changes in the digesta-adherent rumen bacterial communities of dairy cattle grazing pasture. PLoS ONE.

[29] Gharechahi J., Vahidi M.F., Ding X.-Z., Han J.-L., Salekdeh G.H. (2020). Temporal changes in microbial communities attached to forages with different lignocellulosic compositions in cattle rumen. FEMS Microbiol. Ecol..

[30] Lima J., Martínez-Álvaro M., Mattock J., Auffret M.D., Duthie C.-A., Cleveland M.A., Dewhurst R.J., Watson M., Roehe R. (2024). Temporal stability of the rumen microbiome and its longitudinal associations with performance traits in beef cattle. Sci. Rep..

[31] Adeyemi J.A., Peters S.O., De Donato M., Pech-Cervantes A.A., Ogunade I.M. (2020). Effects of a blend of *Saccharomyces cerevisiae-based* direct-fed microbial and fermentation products on plasma carbonyl-metabolome and fecal bacterial community of beef steers. J. Anim. Sci..

[32] Gaffney J., Embree J., Gilmore S., Embree M. (2021). *Ruminococcus bovis* sp. nov., a novel species of amylolytic Ruminococcus isolated from the rumen of a dairy cow. Int. J. Syst. Evol. Microbiol..

[33] Yang J., Li Y., Zhao Y., Xue M., Jin Y. (2025). Understanding the differences in rumen bacteria and their impact on dairy cows’ production performance: A review. Anim. Nutr..

[34] Zhang X., Dong X., Wanapat M., Shah A.M., Luo X., Peng Q., Kang K., Hu R., Guan J., Wang Z. (2022). Ruminal pH pattern, fermentation characteristics and related bacteria in response to dietary live yeast (*Saccharomyces cerevisiae*) supplementation in beef cattle. Anim. Biosci..

[35] Xue Y., Sun H., Guo H., Nie C., Nan S., Lu Q., Chen C., Zhang W. (2024). Effect of the supplementation of exogenous complex non-starch polysaccharidases on the growth performance, rumen fermentation and microflora of fattening sheep. Front. Vet. Sci..

[36] Giraldo L.A., Ranilla M.J., Tejido M.L., Carro M.D. (2007). Influence of exogenous fibrolytic enzymes and fumarate on methane production, microbial growth and fermentation in Rusitec fermenters. Br. J. Nutr..

[37] Söllinger A., Tveit A.T., Poulsen M., Noel S.J., Bengtsson M., Bernhardt J., Frydendahl Hellwing A.L., Lund P., Riedel K., Schleper C. (2018). Holistic assessment of rumen microbiome dynamics through quantitative metatranscriptomics reveals multifunctional redundancy during key steps of anaerobic feed degradation. mSystems.

[38] Humer E., Kröger I., Neubauer V., Schedle K., Reisinger N., Zebeli Q. (2018). Supplementing phytogenic compounds or autolyzed yeast modulates ruminal biogenic amines and plasma metabolome in dry cows experiencing subacute ruminal acidosis. J. Dairy Sci..

[39] Wang D.S., Zhang R.Y., Zhu W.Y., Mao S.Y. (2013). Effects of subacute ruminal acidosis challenges on fermentation and biogenic amines in the rumen of dairy cows. Livest. Sci..

[40] Xue F., Pan X., Jiang L., Guo Y., Xiong B. (2018). GC–MS analysis of the ruminal metabolome response to thiamine supplementation during high grain feeding in dairy cows. Metabolomics.

[41] Dai D., Wang S., Wang X., Gao C., Chai S., Xu X. (2023). High-grain diet feeding altered blood metabolites, rumen microbiome, and metabolomics of yaks. Fermentation.

[42] Kolackova I., Skladanka J., Skalickova S., Horky P., Cernei N., Lackova Z., Trinacty J., Adam V. (2021). Degradation of biogenic amines and in vitro evaluation of ruminal parameters of the ruminal fluid of Charolais sheep. Rev. Bras. Zootec..

[43] Xiang H., Dong X., Lin X., Hou Q., Wang Z. (2025). Effects of yeast culture supplementation on milk yield and milk composition in Holstein dairy cows: A meta-analysis. Animals.

[44] Kalmus P., Orro T., Waldmann A., Lindjärv R., Kask K. (2009). Effect of yeast culture on milk production and metabolic and reproductive performance of early lactation dairy cows. Acta Vet. Scand..

